# Isolation and development of microsatellite loci in an African Woodpecker (*Campethera nivosa*) using polymerase chain reaction and DNA sequencing

**DOI:** 10.1186/s13104-015-1165-1

**Published:** 2015-05-30

**Authors:** Nausheen F Khan, Kellie C Murdoch, Kevin A Feldheim, Ben D Marks, Norbert J Cordeiro

**Affiliations:** Department of Biological and Chemical Sciences, Roosevelt University, 430S Michigan Ave, Chicago, USA; Pritzker Laboratory for Molecular Systematics and Evolution, Integrative Research Center, Field Museum, 1400 S Lake Shore Dr, Chicago, USA; Gantz Family Collections Center, Field Museum, 1400 S Lake Shore Dr, Chicago, USA

**Keywords:** Microsatellite, *Campethera nivosa*, Congo River, Lowland rainforest

## Abstract

**Background:**

The Buff-spotted Woodpecker (*Campethera nivosa*) is a resident bird species that is distributed in lowland rainforest habitats from western to eastern Africa. We developed species-specific microsatellite markers to examine the population genetics of this species.

**Findings:**

Twenty-one microsatellite loci were isolated from *C. nivosa*. Of these, 15 were found to amplify consistently. These loci were then tested for variability in 15 individuals from different lowland forest localities. The number of alleles ranged from 3 to 13 per locus, with observed and expected heterozygosity ranging from 0.100 to 0.917 and 0.485 to 0.901, respectively. Four loci exhibited significant heterozygote deficiency while one had an excess of heterozygotes. None of the loci exhibited linkage disequilibrium.

**Conclusion:**

These polymorphic microsatellite markers will be used to study genetic variability in populations of *C. nivosa* across either sides of the Congo River to evaluate the effect of the river as a barrier to gene flow.

## Findings

The Buff-spotted Woodpecker (*Campethera nivosa*) is a resident (non-migratory) bird of the African lowland rainforests [1]. This species is not currently threatened, and the population is classified as “stable” on the Red List [2]. Despite its widespread distribution little is known about patterns of molecular geographic variation in this species. Large rivers, like the Congo and its tributaries can act as barriers to dispersal for various species of birds, monkeys, apes, and rodents [3–5]. Here, we develop species-specific microsatellite markers for *C. nivosa* which can be used to better understand the genetic diversity and population structure of this species [6].

Genomic DNA (gDNA) was extracted from preserved liver, muscle, and heart tissues of 15 individuals from various lowland rainforest localities (Uganda, Democratic Republic of Congo, Ghana, Gabon, Central African Republic) using the DNeasy® Blood and Tissue kit following the manufacturer’s protocol (QIAGEN Inc. Valencia, CA). Microsatellite markers were isolated using an enrichment protocol [7]. Genomic DNA from one individual was digested using RsaI and XmmI (New England Biolabs). Following digestion, 100 μL each of 10 μM SuperSNX24 and 10 μM SuperSNX24 + 4p primers (FOR: 5′-GTTTAAGGCCTAGCTAGCAGAATC and REV: 5′-GATTCTGCTAGCTAGGCCTTAAACAAAA) were ligated onto the fragmented DNAs. Biotinylated dinucleotide [(TG)_12_, (AG)_12_] and tetranucleotide [(AGAT)_8_, (AAAT)_8_, (ACAT)_8_, (AAGT)_8_, (AACT)_8_] probes were hybridized to gDNA to capture DNA fragments with repetitive elements. These fragments were isolated using streptavidin-coated magnetic beads (Dynabeads M-280 Invitrogen, Carlsbad, CA) in the presence of a magnetic field. The bead-probe complex was washed twice using 2× SSC (saline-sodium citrate buffer) and 0.1% SDS (sodium dodecyl sulfate) solution and four times using 1× SSC, 0.1% SDS at 53°C. The enriched DNA was precipitated with 3 M sodium acetate and 95% ethanol. Enriched fragments were amplified using a recovery PCR. This was performed in a 25 μL reaction containing 1× PCR buffer (10 mM Tris–HCl, 50 mM KCl, pH 8.3), 1 mg/mL BSA, 1.5 mM MgCl_2_, 0.16 mM of each dNTP, 0.52 μM of SuperSNX-24 and 1U Taq polymerase under the following cycling conditions: 95°C for 2 min; 25 cycles of 95°C for 20 s, 60°C for 20 s, 72°C for 1.5 min; 72°C for 30 min. PCR products were cloned using the TOPO TA Cloning® Kit (Invitrogen, Carlsbad, CA). The resultant bacterial colonies with inserts (genomic DNA) were used as template for PCR containing 1× PCR buffer (10 mM Tris–HCl, 50 mM KCl, pH 8.3), 1.5 mM MgCl_2_, 1 mg/mL BSA, 0.12 mM of each dNTP, 0.25 μM of the universal M13 primers, and 1U *Taq* polymerase. Thermal cycling proceeded as follows: 95°C for 10 min, followed by 25 cycles of 95°C for 20 s, 50°C for 20 s, and 72°C for 90 s. These PCR products were cleaned using ExoSAP-IT® following the manufacturer’s protocol (Affymetrix, Santa Clara, CA). Cycle sequencing was performed using the Big Dye Terminator v 3.1 Cycle Sequencing Kit (Applied Biosystems, Foster City, CA), and sequences were run on a 3730 DNA Analyzer. A total of 240 sequences were isolated and manually checked for the presence of repeats and from these, 21 (8.8%) primer sets were developed using Primer3 [8, 9].

Genotyping PCR for individuals loci were performed in 10 μL reactions using 1× PCR buffer (10 mM Tris–HCl, 1.5 mM MgCl_2_, 50 mM KCl, pH 8.3), 0.16 μM of fluorescently labeled universal M13 primer and the species-specific reverse primer, 0.04 μM of the species-specific forward primer with a 5′-M13 tail [10], 0.20 mM each dNTP, 1 unit Taq and 40 ng genomic DNA was run at following conditions: 94°C for 4 min, 30 cycles of 94°C for 30 s, Ta (Table 1) for 30 s, 72°C for 45 s, 8 cycles of 94°C for 30 s, 53°C for 30 s, 72°C for 45 s, and 72°C for 10 min. Fluorescently labeled PCR products were run with an internal size standard (GeneScan™ 500® LIZ, Applied Biosystems, Foster City, CA) on a 3730 DNA Analyzer, and amplicons were sized using GENEMAPPER v3.7. Number of alleles and observed (Ho) and expected (He) heterozygosities were calculated using GenAlEx® software [11, 12]. Tests for heterozygote deficit and excess and linkage disequilibrium were done using GENEPOP® version 4.2 [13, 14]. Probability of identity was calculated for individual loci and across all loci using GenAlEx [15].

**Table 1:** Characteristics for 15 polymorphic microsatellites in the African Buff-spotted Woodpecker *Campethera nivosa*

Locus	Primer sequence (5′–3′)	Primer labeling	Repeat motif	Ta (°C)	n	HWE	k	Ho	He	PID	GenBank accession no.
CNI 1*	F: TGTAAAACGACGGCCAGTGGTGGTGGAGTCACCTTCAT	VIC	(GTATT)7	60	15	1	3	0.917	0.531	3.2E−01	KP418965
	R: GTGTCTTCCTTTACTTGCCCTTCTTGC										
CNI 2*	F: TGTAAAACGACGGCCAGTCCCCTCTTGGAAGTGTTCAA	VIC	(AAAC)5	60	15	<0.0001	5	0.1	0.485	2.9E−01	KP636531
	R: GTGTCTTGGGGGAGTTTGACTCAAGTG										
CNI 3*	F: TGTAAAACGACGGCCAGTAAAGACATCCATTGCCCTTG	6-FAM	(AGACT)12	59	15	<0.0001	7	0.25	0.747	9.6E−02	KP636532
	R: GTGTCTTTCTTCCAACCTGGTCTGGTC										
*CNI 4*	F: TGTAAAACGACGGCCAGTAGACTGGATGGGACACTTGG	VIC	(ATTTCT)11	50	15	0.7509	8	0.818	0.752	9.2E−02	KP418966
	R: GTGTCTTAGTGGCACCTCCTGAGACAT										
*CNI 6*	F: TGTAAAACGACGGCCAGTGCAAAAGGTGGTATTGGAAGA	VIC	(AC)6	60	15	0.3507	4	0.667	0.705	1.4E−01	KP418967
	R: GTGTCTTTGTGTGCTGGAATAGGCAAG										
*CNI 7*	F: TGTAAAACGACGGCCAGTATTTTCCCCCGTCTCTGATT	6-FAM	(TG)6	54	15	0.0072	4	0.273	0.624	1.9E−01	KP418968
	R: GTGTCTTCAAACGAACATCACCACCAC										
CNI 8*	F: TGTAAAACGACGGCCAGTTGGATGATAGGTTGGACGTG	VIC	(CTATT)10	59	15	<0.0001	8	0.444	0.852	3.9E−02	KP636533
	R: GTGTCTTGCCCATCAACAGAAAGCAGT										
*CNI 9*	F: TGTAAAACGACGGCCAGTCCTCCTCTAACACCACACCA	VIC	(CA)10	59	15	0.0188	12	0.75	0.889	2.2E−02	KP418969
	R: GTGTCTTGACCAGGCCAGTGGATTTTA										
*CNI 11*	F: TGTAAAACGACGGCCAGTTGGCTCCACACTGAGTTGTC	VIC	(AATAG)12	60	15	0.4846	10	0.917	0.847	4.0E−02	KP418970
	R: GTGTCTTCGAAGGTCTCTTCCAACCTG										
*CNI 12*	F: TGTAAAACGACGGCCAGTACAGCCTCTCCCATTGTCTC	NED	(AGAAT)5	50	15	0.0482	3	0.455	0.632	2.1E−01	KP418971
	R: GTGTCTTGCTTGGTGCCATGGTTTAGT										
*CNI 13*	F: TGTAAAACGACGGCCAGTTTCCAACCTGGTCAATTCAA	VIC	(CTATT)11, (CTACT)10	57	15	0.005	13	0.636	0.901	1.8E−02	KP418972
	R: GTGTCTTGGCATGCCTAGCTTTGGATA										
*CNI 15*	F: TGTAAAACGACGGCCAGTTCTTCCTAGGGCCTGTCACT	NED	(TCTA)7	59	15	0.0034	10	0.667	0.84	4.4E−02	KP418973
	R: GTGTCTTTCCACTTGAAAGGAAAGAGGTC										
*CNI 16*	F: TGTAAAACGACGGCCAGTTTTGACCAAGGAGGGAAAAA	NED	(GATA)8	59	15	0.9271	7	0.75	0.698	1.2E−01	KP418974
	R: GTGTCTTCAGGGGATTATAGGGGATGG										
*CNI 17*	F: TGTAAAACGACGGCCAGTTGGAAGACTGGGACCAAAAC	NED	(ATCT)12	59	15	0.398	10	1	0.865	3.3E−02	KP418975
	R: GTGTCTTGAATAATCACAATTGTTAATCTGCAT										
*CNI 18*	F: TGTAAAACGACGGCCAGTTGGAAGACTGGGACCAAAAC	VIC	(CTAT)12	59	15	0.7442	8	0.833	0.785	7.1E−02	KP418976
	R: GTGTCTTGAATAATCACAATTGTTAATCTGCAT										

*Ta* optimized annealing temperature, *n* number of individuals genotyped, *k* number of alleles, *Ho* observed heterozygosity, *He* expected heterozygosity, *HWE* p values from heterozygote deficit tests, *PID* probability of identity.

* Departure from Hardy–Weinberg equilibrium (in terms of heterozygote deficit) following Bonferroni correction. CNI1 exhibited heterozygote excess (p = 0.01).

Fifteen primer pairs were developed from a total of 21 tested on 15 *C. nivosa* individuals. The observed and expected heterozygosity ranged from 0.100 to 0.917 and 0.485 to 0.901, respectively (Table 1). After applying Bonferroni correction [16], CNI2, CNI3 and CNI8 exhibited departure from Hardy–Weinberg equilibrium, in terms of heterozygote deficit, while CNI1 exhibited a significant excess of heterozygotes. Probability of identity for each locus is shown in Table 1; the cumulative probability of identity for these loci was 3.7 × 10^−17^. These markers will be used to evaluate population genetic structure of *C. nivosa*.

### Availability of the supporting data

All the microsatellite sequences in this paper were deposited in the National Centre for Biotechnology Information (http://www.ncbi.nlm.nih.govwebcite). They are now accessible via the GenBank accession numbers KP418965–KP418976 and KP636531–KP636533.

## References

[1] Fry CH, Keith S, Urban EK (1988). The birds of Africa.

[2] The IUCN Red List of Threatened Species. Version 2014.2 (2012). http://www.iucnredlist.org. Accessed 9 Aug 2014

[3] Kennis J, Nicolas V, Hulselmans J, Katuala PG, Wendelen W, Verheyen E (2011). The impact of the Congo River and its tributaries on the rodent genus *Praomys*: speciation origin or range expansion limit. Zool J Linn Soc.

[4] Voelker G, Marks BD, Kahindo C, A’genonga U, Bapeamoni F, Duffie LE (2013). River barriers and cryptic biodiversity in an evolutionary museum. Ecol Evol.

[5] Marks BD (2010). Are lowland rainforests really evolutionary museums? Phylogeography of the green hylia (*Hylia prasina*) in the Afrotropics. Mol Phylogenet Evol.

[6] Presti F, Wasko A (2014). A review of microsatellite markers and their application on genetic diversity studies in parrots. Open J Genet.

[7] Glenn TC, Schable NA (2005). Isolating microsatellite DNA loci. Methods Enzymol.

[8] Untergrasser A, Cutcutache I, Koressaar T, Ye J, Faircloth BC, Remm M (2012). Primer3—new capabilities and interfaces. Nucleic Acids Res.

[9] Koressaar T, Remm M (2007). Enhancements and modifications of primer design program Primer3. Bioinformatics.

[10] Schuelke M (2000). An economic method for the fluorescent labeling of PCR fragments. Nat Biotechnol.

[11] Peakall R, Smouse PE (2012). GenAlEx 6.5: genetic analysis in Excel. Population genetic software for teaching and research-an update. Bioinformatics.

[12] Peakall R, Smouse PE (2006). GENALEX 6: genetic analysis in Excel. Population genetic software for teaching and research. Mol Ecol Notes.

[13] Raymond M, Rousset F (1995). GENEPOP (version 1.2): population genetics software for exact tests and ecumenicism. J Hered.

[14] Rousset F (2008). Genepop’007: a complete reimplementation of the Genepop software for Windows and Linux. Mol Ecol Resour.

[15] Peakall R, Smouse PE (2006). GENALEX 6: genetic analysis in Excel. Population genetic software for teaching and research. Mol Ecol Notes.

[16] Rice WR (1989). Analyzing tables of statistical tests. Evolution.

